# Advances in neoantigen-based immunotherapy for head and neck squamous cell carcinoma: a comprehensive review

**DOI:** 10.3389/fonc.2025.1593048

**Published:** 2025-05-15

**Authors:** Manzhu Jiang, Jiefu Li, Jianhua Wei, Xuerong Yang, Weiqi Wang

**Affiliations:** ^1^ College of Life Sciences, Shandong Agricultural University, Tai’an, China; ^2^ Guangzhou National Laboratory, Guangzhou, China; ^3^ State Key Laboratory of Oral & Maxillofacial Reconstruction and Regeneration, National Clinical Research Center for Oral Diseases, Shaanxi Key Laboratory of Stomatology, Department of Prosthodontics, School of Stomatology, The Fourth Military Medical University, Xi’an, China

**Keywords:** head and neck squamous cell carcinoma (HNSCC), neoantigen, personalized neoantigen vaccines, immune checkpoint inhibitors (ICIs), combination therapy

## Abstract

Head and Neck Squamous Cell Carcinoma (HNSCC), ranking among the six most prevalent malignancies worldwide, is characterized by significant heterogeneity. Conventional monotherapeutic approaches, including surgical intervention, radiotherapy, and chemotherapy, often fail to achieve complete tumor cell elimination, consequently leading to disease recurrence and metastatic progression. In this context, personalized immunotherapeutic strategies, particularly cancer vaccines and immune checkpoint inhibitors, have emerged as promising therapeutic modalities for patients with recurrent/metastatic (R/M) HNSCC. Neoantigens, which exhibit selective expression in tumor tissues while remaining absent in normal tissues, have garnered considerable attention as novel targets for HNSCC personalized immunotherapy. However, the marked heterogeneity of HNSCC, coupled with patient-specific HLA variations, necessitates precise technical identification and evaluation of neoantigens at the individual level-a significant contemporary challenge. This comprehensive review systematically explores the landscape of neoantigen-based immunotherapy in HNSCC, including neoantigen sources, screening strategies, identification methods, and their clinical applications. Additionally, it evaluates the therapeutic potential of combining neoantigen-based approaches with other immunotherapeutic modalities, particularly immune checkpoint inhibitors, providing valuable insights for future clinical practice and research directions in HNSCC treatment.

## 1 Introduction

**Figure 1:**
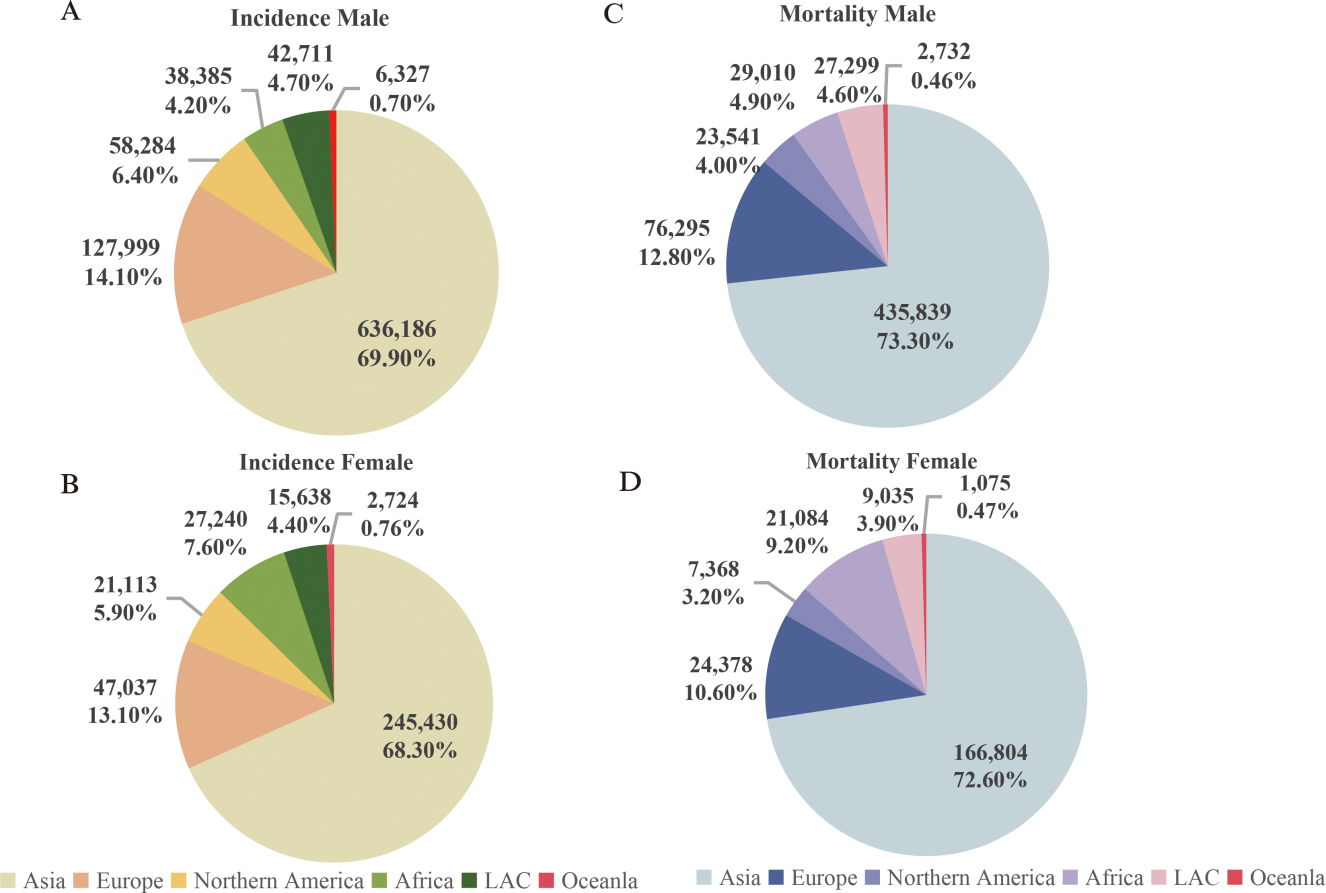
Incidence and mortality rates of head and neck cancer (Updated 2022). **(A)** Incidence rate of head and neck cancer in males; **(B)** Incidence rate of head and neck cancer in females; **(C)** Mortality rate of head and neck cancer in males; **(D)** Mortality rate of head and neck cancer in females. Note: Source: https://gco.iarc.fr/today.

According to the data released by the World Health Organization (WHO) in 2022, head and neck cancer ranks as the sixth most common malignant tumor globally (1). It exhibits a relatively high incidence and mortality rate in Asia, and both rates being notably higher in men (**Figures 1A, C**) compared to women (**Figures 1B, D**) (source: https://gco.iarc.fr/today). Head and neck cancer encompasses a group of malignant tumors that arise in the epithelial tissues of the paranasal sinuses, lips, oral cavity, nasal cavity, pharynx, and larynx (2). The most common histological subtype is squamous cell carcinoma, which accounts for approximately 90% of cases (3). The incidence of HNSCC continues to rise and is projected to increase by 30% by 2030, leading to an estimated 1.08 million new cases annually (4).

Head and Neck Squamous Cell Carcinoma (HNSCC) can be induced by various factors, including chronic excessive alcohol consumption, poor oral hygiene, viral infections, betel nut chewing, and smoking (5). The complex anatomical and physiological structures of the head and neck contribute to highly heterogeneous nature of HNSCC (6). The majority of patients are diagnosed at the locally advanced stages of the disease (7). Conventional treatment modalities for HNSCC include surgery, chemotherapy, and radiotherapy (8). However, these treatments are often associated with severe late-stage adverse effects, such as renal impairment, hearing loss, myelosuppression, and aspiration pneumonia (9). A significant shift in treatment strategies involves the combination of immune checkpoint inhibitors, such as anti-PD-1 monoclonal antibodies, with chemotherapy. This approach has led to improvements in the survival rates of patients with recurrent or metastatic (R/M) HNSCC (10). Despite these advances, the four-year survival rate remains disappointingly low, ranging from only 15% to 19% (11).

HNSCC demonstrates significantly heterogeneity in terms of molecular characteristics, cellular phenotypes, and the composition of the tumor microenvironment (TME) (12, 13). For instance, the distribution of cancer-associated fibroblasts (CAFs) and the expression of various CAF markers exhibit notable variations among individual HNSCC patients (14). Additionally, there are differences in the mutational burden between human papillomavirus-positive (HPV+) and human papillomavirus-negative (HPV−) tumors (12). These factors collectively influence the outcome of conventional clinical treatments. Therefore, the development of more effective targeted therapies is essential to improving the prognosis for HNSCC patients.

Personalized immunotherapy is rapidly reshaping the treatment landscape of HNSCC. Neoantigens play a central role in tumor vaccines and immune checkpoint blockade (ICB) strategies (15). Tumor antigens can be categorized into two types. The first category, known as tumor-associated antigens (TAAs), are highly expressed in tumor tissues while exhibiting low expression in normal tissues. The second category, tumor neoantigens (also referred to as tumor-specific antigens, or TSAs), consists of unique peptide segments generated by genomic aberrations, transcriptomic irregularities, abnormal post-translational modifications, and other factors in tumor cells (16, 17). These neoantigens can be recognized by the major histocompatibility complex (MHC) on antigen-presenting cells (APCs), and the resulting tumor-specific peptide-HLA complexes can be identified by T cells, thereby triggering an anti-tumor immune response (18). Immunogenomic techniques have been employed to predict a large number of neoantigens arising from mutations based on cancer genomic data. However, proteomic studies examining peptides binding to HLA have shown that most of the predicted neoantigens were not detected (19). The discrepancy between predicted and observed highlights limitations in the current prediction methods and technologies. To improve the identification of clinically relevant neo-epitopes, epitope prediction should incorporate multiple methods, alongside advanced quality assessment metrics for neo-epitopes.

## 2 Challenges in the treatment of HNSCC

HNSCC exhibits a high degree of both inter-tumor and intra-tumor heterogeneity (20). HPV-positive and HPV-negative HNSCC not only originate from distinct anatomical sites but also display divergent mutation spectra, molecular characteristics, immune landscapes, and clinical prognoses (21). Additionally, HNSCC is a notable immunosuppressive malignancy. For instance, regulatory T cells (Tregs), tumor-associated macrophages, and myeloid-derived suppressor cells (MDSCs) have been shown to enhance immune evasion of HNSCC (22). Human leukocyte antigens (HLAs), which play a central role in the immune response, exhibit considerable genetic polymorphism and vary across HNSCC patients. For example, Tang et al. identified that the genetic susceptibility to nasopharyngeal carcinomas is linked to the HLA haplotype A*0206, a subtype associated with a heightened risk of the disease in East and Southeast Asia (23). Similarly, Makni et al. found that HLA-A31, A33, A19, B16, B53, and the alleles DRB103, DRB113, and DQB1*02 are potential susceptibility genes for nasopharyngeal carcinoma, while HLA-B14, HLA-B35, and DRB1*01, DQB1*05 may offer a protective effects (24). Understanding the HLA genotypes of different HNSCC patients can help elucidate the pathogenesis of the disease and facilitate the development of more precise, targeted therapies.

The primary goal in treating patients with HNSCC are to achieve complete tumor resection or effective tumor control, extend survival, and minimize treatment-related adverse effects (25). For patients with locally advanced or recurrent/metastatic (R/M) HNSCC, surgery is typically the first-line curative approach. In cases where surgery is not feasible, a combination of radiotherapy and cisplatin is commonly used (26). For patients who cannot tolerate cisplatin or are elderly (over 70 years of age), radiotherapy alone is an alternative (27). The U.S. Food and Drug Administration (FDA) has approved the use of nivolumab and pembrolizumab for treating patients HNSCC patients who have developed resistance to platinum-based therapies (28–30). Results from the phase II KEYNOTE-055 study showed that pembrolizumab monotherapy had some efficacy in platinum-resistant HNSCC. The study reported an objective response rate (ORR) of 16%, a duration of response (DOR) of 8 months, progression-free survival (PFS) of 2.1 months, and an overall survival (OS) of 8 months (31). Despite these findings, patients still face challenges such as a relatively low response rate and a high risk of recurrence during treatment.

Patients with HNSCC often present with malnutrition or are complicated by other comorbidities. Traditional treatment methods, while effective in some cases, can cause significant damage to the immune system and trigger severe side effects (32). Additionally, HNSCC is a highly heterogeneous malignancy, and variations in human leukocyte antigen (HLA) genotypes among patients contribute to substantial differences in prognosis (33). Given these challenges, there is an urgent need for continued research to explore alternative strategies for HNSCC (11). This review focuses on the development of personalized treatment regimens based on neoantigens. Such approaches offer promising application potential and substantial clinical value, contributing to more efficient treatment strategies for patients with HNSCC.

## 3 The source of neoantigens

**Figure 2:**
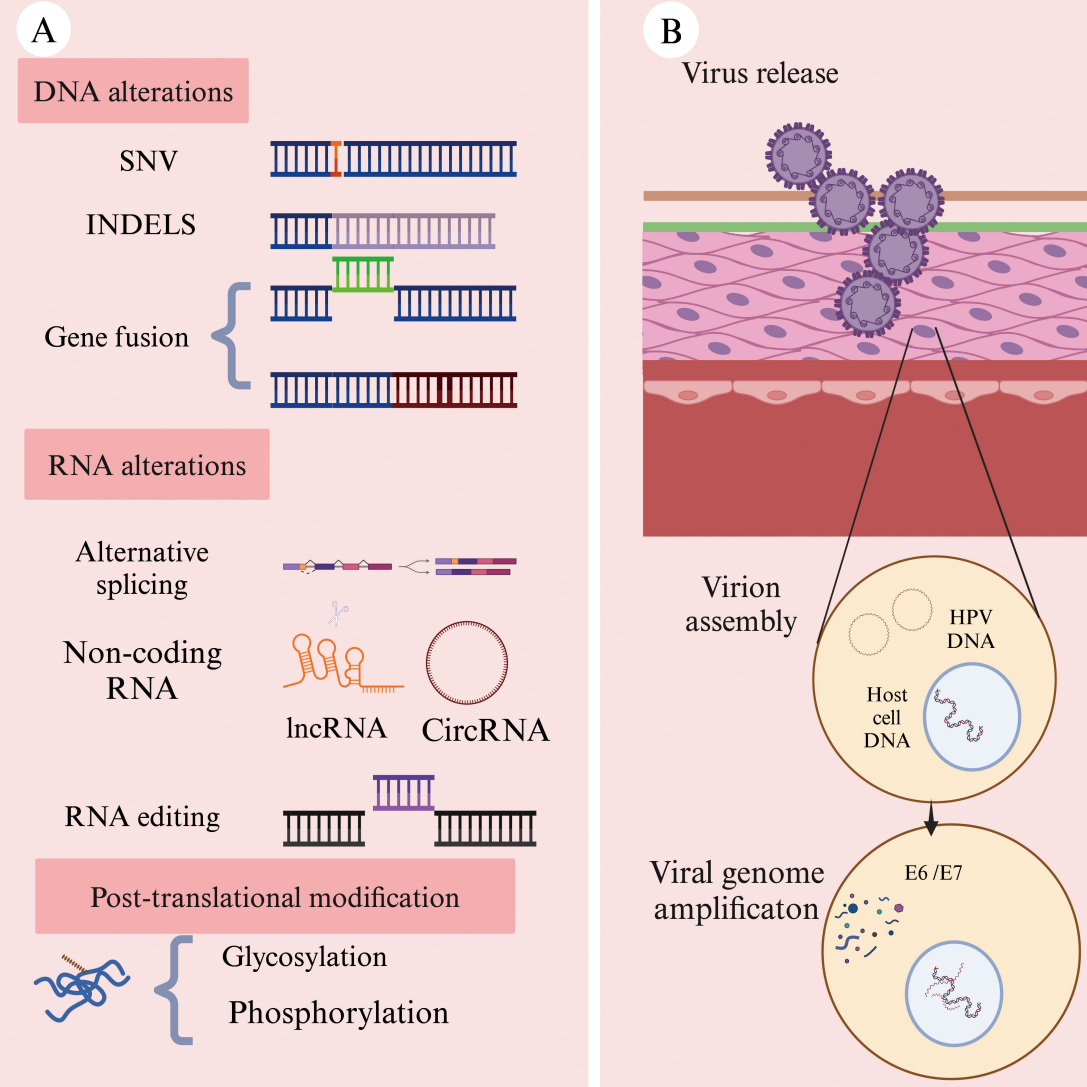
Common sources of neoantigens in HNSCC (Created with BioRender.com). **(A)** Neoantigens can arise from multiple molecular levels: at the genomic level through single-nucleotide variants (SNVs), insertions or deletions (INDELs), and gene fusions; at the transcriptomic level through alternative splicing, polyadenylation (pA), RNA editing, and non-coding regions; and at the proteomic level through dysregulated translation and post-translational modifications (PTMs). **(B)** Oncogenic viruses, such as human papillomavirus (HPV), can integrate their genetic material into the host cell genome. The expression of these viral genes within hose cells may result in the production of tumor-specific neoantigens.

Tumor neoantigens are defined by their exclusive expression in tumor tissues, with no detectable expression in healthy tissues. Additionally, they evade the central tolerance mechanism of T-cells, making them highly immunogenic (34). Due to those properties, neoantigens can elicit a robust anti-tumor immune response and are considered highly promising targets in personalized immunotherapy (35). Neoantigens primarily arise from genomic alterations (**Figure 2A**), including single-nucleotide variants (SNVs), small insertions or deletions (INDELs), and gene fusions. Beyond genetic mutations, additional mechanisms contribute to neoantigen formation, including alternative splicing, aberrant RNA editing (36), non-coding RNA expression (37), and abnormal post-translational modifications (PTMs) of proteins (38) (**Figure 2A**). In HNSCC, viral oncogenes can integrate into the host genome, providing an additional source of neoantigens and further diversifying the tumor antigen landscape (39) (**Figure 2B**).

### 3.1 Non-viral-derived neoantigens

Single-nucleotide variants (SNV) are the most common type of genetic mutation in tumor cells. When a single-nucleotide alteration occurs in DNA, it can affect the coding sequence of the gene, leading to the generation of neoantigens (40). Small insertion or deletions (INDELs) refer to the addition or removal of one or more bases at specific loci in the DNA sequence compared to the reference genome (41). If an INDEL occurs within a gene’s coding region or splice site, it can alter protein structure and function, thereby triggering the production of neoantigens (42).

Compared to SNVs, INDELs can result in the formation of novel open reading frames (ORFs), leading to the synthesis of a large number of non-self peptides (34). Notably, neoantigens generated by INDELs exhibit significantly higher immunogenicity than those derived from SNVs (43). Although INDELs are less frequent than SNVs, the results of a large-scale analysis of 5,777 solid tumors across 19 cancer types in The Cancer Genome Atlas (TCGA) demonstrated that INDEL-derived neoantigens are more immunogenic than those produced by SNVs (44).

Gene fusions represent another important class of mutations. When a gene fusion occurs, it can create open reading frame(ORF) that encodes an entirely new protein (45). For instance, in sinus cancer and nasopharyngeal carcinoma, the DEK-AFF2 fusion gene has been identified. A peptide (DKESEEEVS) derived from DEK-AFF2 has been shown to stimulate the activation of autologous peripheral blood mononuclear cells (PBMCs) in a major histocompatibility complex (MHC)-dependent manner (46). The discovery of fusion gene-derived neoantigens has expanded the repertoire of potential targets for cancer immunotherapy, positioning them as a key driving force in the development of novel therapeutic strategies (47).

RNA alternative splicing involves mutations in cis-acting elements and alterations in trans-acting regulatory factors. Neoantigens derived from this process often exhibit enhanced immunogenicity (48). Cis-acting mutations primarily include intron retention and exon skipping, while trans-acting alterations can induce neoantigen generation across the entire genome (49). Pan-cancer analysis using data from The Cancer Genome Atlas (TCGA) has revealed that somatic mutations in key splicing factors, such as SF3B1 and U2AF1, can lead to the formation of splicing-derived neoantigens across various solid tumors (37). In a study of uveal melanoma patients with SF3B1 mutations, tumor and normal tissue samples were collected to predict splicing variants induced by these mutations. The results demonstrated that the resulting neoantigens exhibited strong immunogenicity (50). Wang et al. applied two computational tools, ScanExitron and ScanNeo, to identify and analyze neoantigens generated through exitron skipping. In this process, exitron are defined as cryptic intronic sequences located within annotated protein-coding exons. Their findings indicated that exitron skipping could produce highly immunogenic neoantigens (51).

Although circular RNAs (circRNAs) do not directly encode proteins, their regulatory functions can influence neoantigen generation. Recent advancements in high-throughput circRNA reporter gene screening and mass spectrometry-based peptidomics workflows have facilitated the identification of circRNAs as a novel source of neoantigens. Zheng et al. successfully demonstrated that circRNAs could induce tumor-specific T cell responses, leading to selective tumor cell elimination (52). This discovery provides new insights and potential therapeutic targets for tumor immunotherapy.

A Phase I clinical trial (NCT06530082) utilized mass spectrometry to analyze human leukocyte antigen class I (HLA-I) in breast cancer samples. This analysis combined with ribosome profiling, successfully identified cryptic antigen peptides generated through atypical translation of circFAM53B, which were capable of binding to HLA-I. The study further evaluated a dendritic cell (DC) vaccine based on CircFAM53B-219aa, administered in combination with camrelizumab for the treatment of HER2-negative advanced breast cancer (53). The results demonstrated that the DC vaccine, designed using circRNA-derivedneoantigens, effectively stimulate T cell activation and induced cytotoxic responses against tumor cells.

Abnormal post-translational modifications (PTM) of proteins can also contribute to the generations of neoantigens. Key modification types include glycosylation, O-linked β-N-acetylglucosamine (O-GlcNAc) modification, and phosphorylation, among others (36). These modifications play a critical role in the shaping neoantigen formation and influencing their immunogenic properties (54).

### 3.2 Virus-derived neoantigens

Viral proteins represent a distinct class of neoantigens in virus-associated tumors, capable of eliciting high-affinity T cell receptor (TCR) responses (55). Certain solid tumors are directly linked to viral infections, such as nasopharyngeal carcinoma, which is associated with caused by Epstein-Barr virus (EBV) infection (56). When viral genes integrate into the host genome, the expression of these foreign genetic elements may lead to the formation of neoantigens, as observed in oropharyngeal cancer caused by human papillomavirus (HPV) infection (57).

High-risk HPV infection accounts for approximately 40% to 70% of head and neck cancers with HPV16 and HPV18 being the most prevalent subtypes (58). The oncoproteins E6 and E7, expressed by high-risk HPV strains, play a pivotal role in disrupting genomic stability and driving tumor progression (59). MEDI0457, a DNA-based immunotherapy, utilizes plasmids encoding interleukin-12 (IL-12) to target the HPV16/18-derived E6 and E7 proteins, demonstrating the potential to elicit a durable anti-tumor immune response (60).

## 4 Identification and prediction of neoantigens

The identification of neoantigens with high immunogenicity is a critical step in developing effective personalized immunotherapies (61). Next-generation sequencing (NGS), also referred to as high-throughput sequencing, is widely used to detect tumor-specific genetic alterations (62). Whole-exome sequencing (WES) and RNA sequencing (RNA-seq) are fundamental tools in genomics research, facilitating the identification of mutations that may give rise to neoantigens (63).

NGS plays a pivotal role in proteogenomics, a field that integrates genomic data, transcriptomic, and proteomic data to enhance cancer research (64, 65). Tretter et al. developed an innovative approach by combining RNA sequencing, proteomic profiling, and whole-exome sequencing to identify neoantigens at the protein level (66). Mass spectrometry (MS) has also advanced the validation of computationally predicted neoantigens, providing a more comprehensive understanding of their immunogenic potential (67).

Genomic approaches primarily predict neoantigens based on DNA and RNA mutation data, making them suitable for the preliminary identification and screening of potential targets (68); In contrast, MS focuses on directly detecting and characterizing MHC-bound polypeptides, offering a reliable method for confirming neoantigens at the proteomic level (69). The integration of these methodologies enhances the accuracy of neoantigen identification, ultimately facilitating the development of targeted cancer immunotherapies.

The T cell receptor (TCR) exhibits remarkable diversity and is capable of recognizing and specifically binding to neoantigen peptides presented by MHC molecules (70). This recognition primarily occurs through specific interactions with complementarity-determining region 1 (CDR1) and 2 (CDR2) (71). Traditional methods for studying TCRs are often based on bulk cell population analysis, which limits their ability to accurately reflect the TCR expression profile of individual T cells.

High-throughput single-cell RNA sequencing (scRNA-seq) has emerged as a powerful technique for studying gene expression at the individual level within a heterogeneous population (72). By applying scRNA-seq to T cells in tumor samples, researchers can identify multiple neoantigen-specific TCRs with high specificity and affinity, capable of precisely recognizing and binding to neoantigens (73). For instance, scRNA-seq libraries were constructed from nasopharyngeal carcinoma (NPC) patient samples and subsequently sequenced using a high-throughput sequencing platform. In-depth analysis of the sequencing data revealed significant clonal expansion of T cells in EBV-positive tumor samples (74). This finding suggests that the sequence characteristics of these TCRs are closely associated with the EBV-derived neoantigens. Despite its advantages, scRNA-seq has certain limitations. One major challenge is maintaining cell integrity and viability throughout the process, as these factors are critical for ensuring accurate single-cell analysis (75). Addressing these challenges will be essential for further advancing the application of scRNA-seq in neoantigen research and personalized immunotherapy.

**Figure 3:**
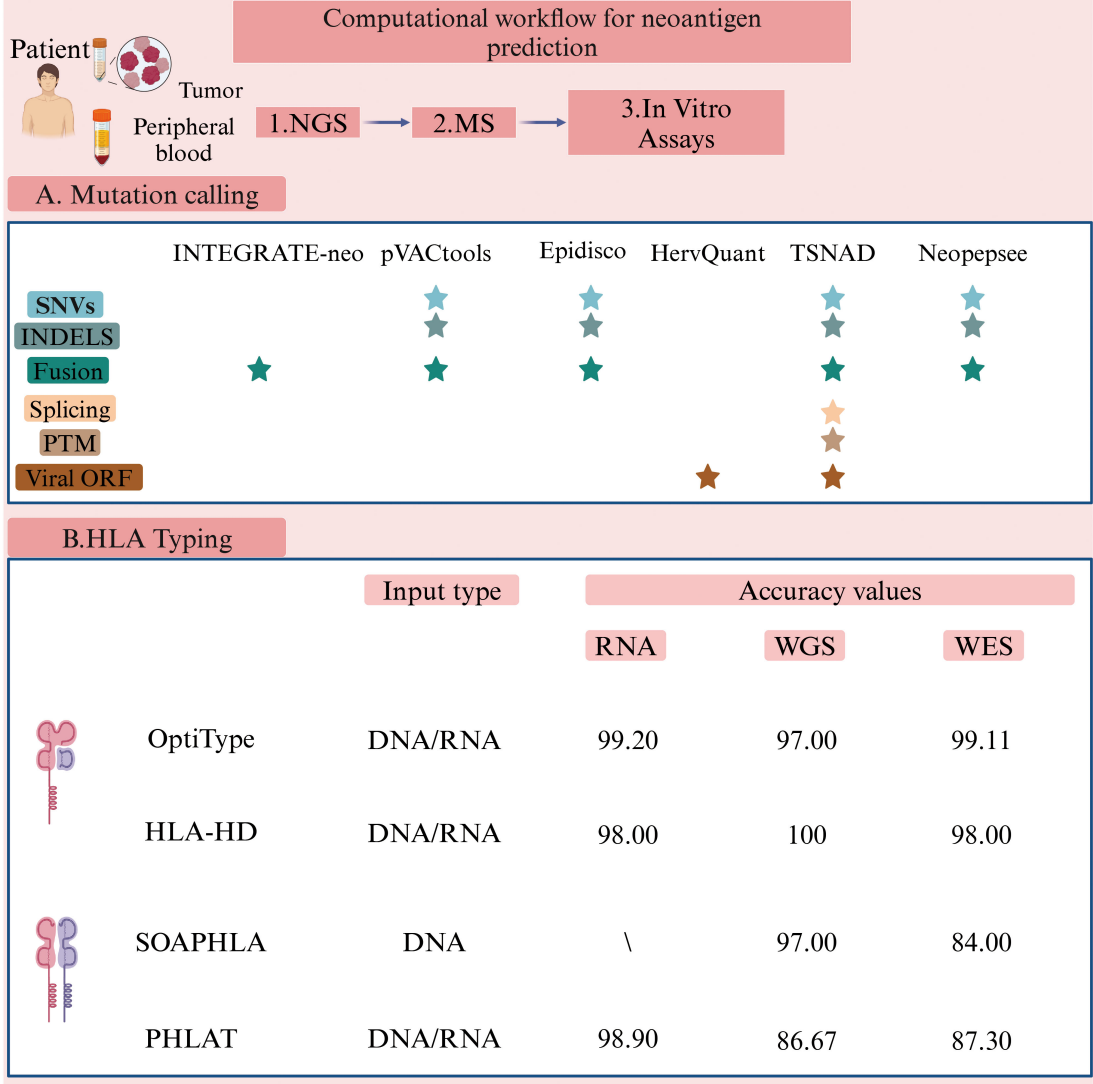
Computational workflow for neoantigen prediction (Created with BioRender.com). Various software packages are utilized to identify sequence variations between tumor and normal cells. These tools predict and prioritize antigen immunogenicity, thus facilitating the selection of optimal tumor neoantigens for therapeutic application. The pVACtools suite consists of multiple functional modules, including pVACseq (personalized Variant Antigens by Cancer Sequencing) for predicting neoantigens derived from somatic mutations, and pVAC-fuse, which identifies neoantigens originating from gene fusions (151) (http://www.pvactools.org). INTEGRATE-Neo is an open-source pipeline designed to utilize NGS data for identifying neoantigens arising from gene fusions, thereby expanding the scope of neoantigen discovery (152) (https://github.com/ChrisMaherLab/INTEGRATE-Neo). Epidisco serves primarily as a workflow orchestration tool, coordinating the parallel execution of analytical processes such as variant detection and neoantigen prediction. It ensures efficient utilization of computational resources, thus accelerating vaccine development (153). HervQuant is specifically designed to analyze and quantify human endogenous retroviruses (hERVs) expression, potentially identifying novel targets for tumor vaccines or immunotherapy (154). TSNAD (Tumor-Specific Neoantigen Detector) follows Genome Analysis Toolkit (GATK) best practice for detecting somatic mutations in cancers and predicting potential neoantigens (155) (https://github.com/jiujiezz/tsnad). Neopepsee integrates sequence features and amino acid immunogenicity profiles through machine learning algorithms to enhance the accuracy and specificity of neoantigen prediction (156) (http://sourceforge.net/projects/neopepsee/).

The human leukocyte antigen (HLA) system, located on the short arm of chromosome 6, is equivalent to the MHC in humans (76). Humans possess more than 24,000 different alleles of HLA class I (HLA-I, including HLA-A, HLA-B, and HLA-C) and HLA class II (HLA-II, including HLA-DR, HLA-DQ, and HLA-DP), with their combined effects leading to significant polymorphism (77). Wichmann et al. have conducted a study on patients with HNSCC presenting distinct HLA characteristics, observing that disease progression varied among these patients based on their HLA profiles (78). Additionally, previous studies have indicated associations between specific HNSCC subtypes and HLA genotypes (79). As a result, determining the HLA genotype of HNSCC patients is an essential step in the neoantigen prediction process (80). Predicting the binding affinity of HLA class II (HLA-II) is more challenging compared to HLA class I (HLA-I). HLA-I molecules present shorter peptide sequences (8–11 amino acids), while HLA-II molecules present longer sequences (11–20 amino acids or even longer) due to their open peptide-binding grooves (81). Consequently, most studies primarily focus on tumor vaccines that utilize antigenic peptides presented by HLA-IA molecules (82). However, the expression of HLA-IA is often downregulated in tumors, which can promote immune evasion by the tumor (83). In contrast, the expression level of HLA-IB is associated with prognosis and immune infiltration (84). When HLA-IA expression is downregulated, peptides that bind to HLA-IB can emerge as highly promising therapeutic targets. Machine learning and deep learning techniques are increasingly used to predict peptide-HLA binding affinities by analyzing the binding sites of neoantigen peptide sequences and HLA molecules (85). A high binding affinity indicates that the mutated peptide is more likely to bind to HLA and be recognized by T cells (86). Several tools have been developed to identify HLA-I alleles, including OptiType (87) and HLA-HD (88), and tools for HLA-II alleles, such as SOAPHLA (89) and PHLAT (90) (**Figure 3**). These tools demonstrate prediction accuracies as high as 99% when compared to HLA-specific typing methods used in clinical practice (91). Among them (**Figure 3**), OptiType currently reports the highest accuracy.

However, HLA-II typing algorithms still require further development to improve their predictive accuracy compared to HLA-I typing algorithms.

Relying solely on algorithmic predictions to screen candidate neoantigens may lead to false-positive results and fail to accurately predict the binding affinities of all possible HLA alleles to peptides (62). Therefore, functional verification is a essential to ensure the efficacy of candidate neoantigens. This process typically involves testing whether candidate peptides can stimulate T-cell proliferation or induce immune responses such as cytokine production *in vitro*, as well as evaluating whether the peptides can provoke effective anti-tumor immune responses *in vivo* (92). One of the key techniques for assessing the immunogenicity of neoantigens is the enzyme-linked immunospot (ELISPOT) assay. This method detects changes in cytokine secretion by T cells after stimulation by neoantigens, using specific antibodies (93). In this assay, patient-derived T cells are co-cultured with dendritic cells (DCs) loaded with candidate neoantigen peptides (94). By measuring the release of cytokines (such as IFN-γ) or the upregulation of activation markers (e.g., CD25), researchers can assess whether T cells are effectively activated (95). Cytotoxicity assays provide another critical approach to evaluate the immunogenicity of neoantigens. In these assays, tumor cell lines expressing patient-specific neoantigens serve as target cells. Patient-derived T cells are co-cultured with the target cells, and subsequent death of the target cells is monitored. This directly reflects the cytotoxic capacity of T cells against tumor cells *in vitro* and is serve as a valuable measure of neoantigens immunogenicity (96).

## 5 Neoantigen-based treatment for HNSCC

Identifying the optimal combination of various immunotherapies is crucial for the effective treatment of recurrent and metastatic (R/M) HNSCC (6). A search using the keywords “Head and neck cancer” and “neoantigen” to search on ClinicalTrials.gov has revealed several registered clinical trials targeting neoantigens for treating head and neck cancer. These trials explore the use of neoantigen vaccines, both in combination with and without immune checkpoint inhibitors (ICIs). A summary of these clinical trials is provided in **Table 1** (Source: https://clinicaltrials.gov/, update on February 20^th^, 2025).

**Table 1:** Neoantigen-based clinical trials for “head and neck cancer” registered on ClinicalTrials.gov.

NCT number	Trial phase	Research status	Tumor type	Deadline	Therapy	Co-administration	Unites	Subject
NCT04266730	1	Not recruiting	HNSCC	2033-06-01	PANDA-VAC(peptide-vaccine)	PD-L1 inhibitor	A	18 Years and older (Adult, Older Adult)
NCT05269381	2	Recruiting	HNSCC	2026-02-24	Peptide-vaccine	PD-L1 inhibitor	B	18 Years and older (Adult, Older Adult)
NCT03552718	1	Active, not recruiting	HNSCC	2025-12-30	YE-NEO-001(yeast-based vaccine)	NA	C	18 Years and older (Adult, Older Adult)
NCT06675201	2	Recruiting	ESCC	2027-10-01	DC-vaccine	ICIs	D	18–80 Years (Adult, Older Adult)
NCT05317325	1	Unknown status	ESCC	2024-04-01	DC-vaccine	NA	D	18–80 Years (Adult, Older Adult)
NCT05192460	NA	Recruiting	EC	2025-06	PGV002(mRNA-vaccine)	PD-1/L1 inhibitor	E	18–75 Years (Adult, Older Adult)
NCT05307835	1	Recruiting	EC	2025-12-03	iNeo-Vac-P01(peptide-vaccine)	NA	F	18–80 Years (Adult, Older Adult)
NCT03908671	NA	Recruiting	EC	2025-12-31	mRNA-vaccine	NA	G	18–75 Years (Adult, Older Adult)
NCT04001413	2	Withdrawn	OC	2021-03-25	DNA-vaccine	ICIs	H	18 Years and older (Adult, Older Adult)

HNSCC, Head and neck squamous cell carcinoma; EC, Esophageal cancer; TIL, Tumor infiltrating lymphocyte; A: UNC Lineberger; B: Mayo Clinic; C: NantBioScience; D: Sichuan University; E:The Affiliated Hospital of the Chinese Academy of Military Medical Sciences; F: Zhejiang University; G: Stemirna Therapeutics; H: KCC; NA, Not available; LS, Lip SCC; OCC, Oral Cavity Cancer; OC, Oropharynx Cancer; LC, Larynx Cancer; HC, Hypopharynx Cancer; ESCC, Esophageal squamous cell carcinoma; ICIs, Immune Checkpoint Inhibitors.

### 5.1 Tumor vaccines targeting neoantigens

**Figure 4:**
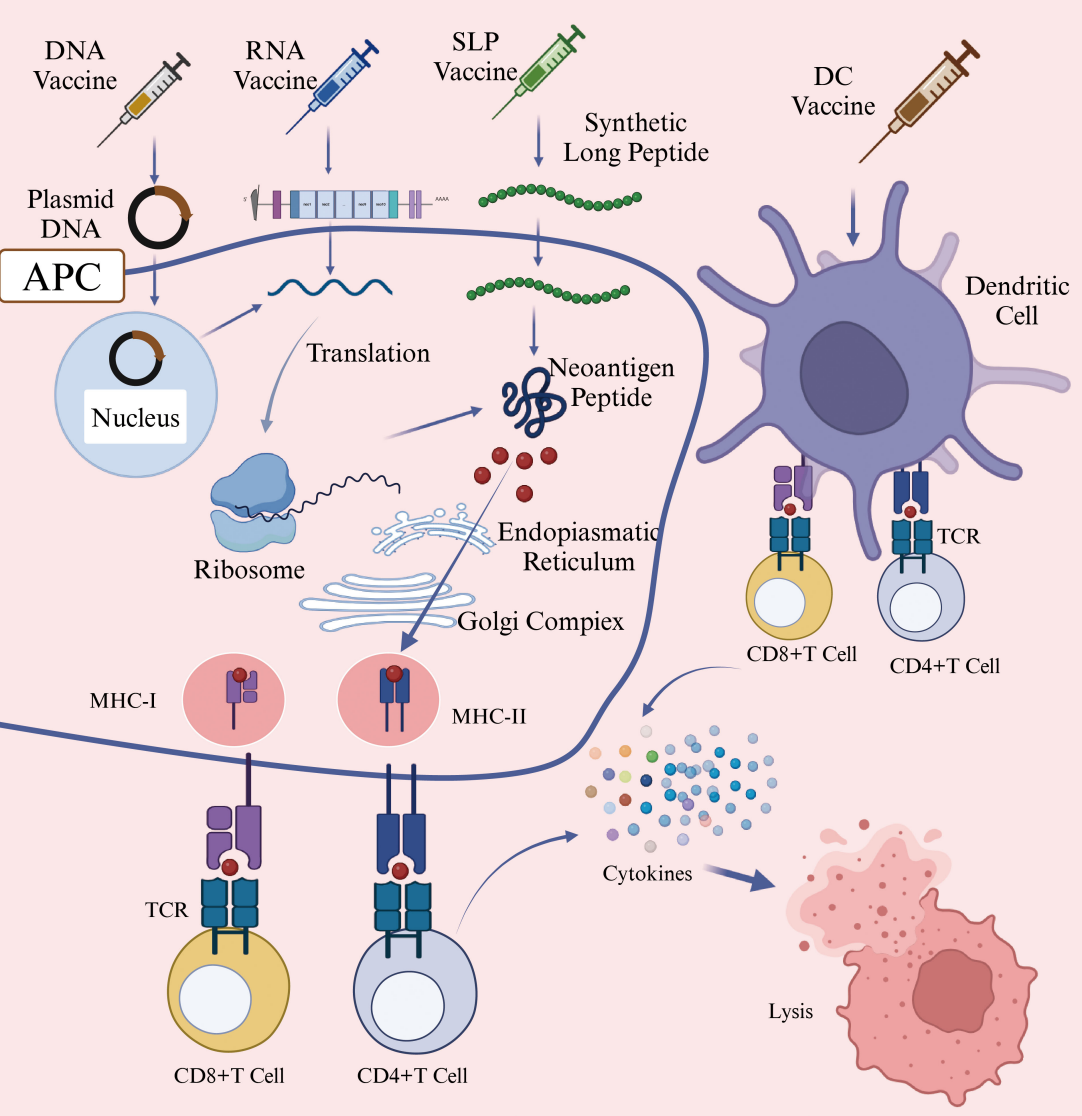
Mechanism of Action of DNA/RNA/SLP/DC Vaccines (Created with BioRender.com). This figure illustrated the mechanisms through which various neoantigen vaccines stimulate immune responses against tumor cells. DNA vaccines introduce genes encoding neoantigens into host cells, leading to neoantigen protein synthesis. RNA vaccines deliver neoantigen-encoding mRNA into cytoplasm, where cellular machinery translates the mRNA directly into neoantigen proteins. Synthetic long peptide (SLP) vaccines consist of multiple neoantigen peptide sequences and are directly internalized by antigen-presenting cells (APCs). Dendritic cell (DC) vaccines involve the ex vivo loading of neoantigens onto DC cells, followed by their reinfusion into patients. Subsequently, APCs process these neoantigens and present peotide fragments via major histocompatibility complex (MHC) molecules to T cells. Activated T cells recognize these presented neoantigens, resulting in targeted immune responses and the elimination of tumor cells. Abbreviations: APC, antigen-presenting cell; DC, Dendritic Cell; MHC-I, major histocompatibility complex class I; MHC-II, major histocompatibility complex class II; TCR, T-Cell receptor; SLP, synthetic long peptide.

Given the high heterogeneity of HNSCC, patients with this malignance may benefit from personalized neoantigen-based vaccines tailored to their specific tumor mutations (97). Although these vaccines are in the early stage of development, they have demonstrated significant potential in cancer immunotherapy. Neoantigen-based tumor vaccines can be broadly categorized into nucleic acid vaccines, peptide vaccines, and dendritic cell (DC) vaccines (**Figure 4**) (98). Each vaccine type utilizes distinct antigen-presenting cell (APC) processing pathways. For DNA vaccines, the genetic material is internalized into the cytoplasm, where it undergoes transcription and translation before being processed into antigenic peptides. For RNA vaccines, translation occurs directly on cytoplasm ribosomes, followed by proteasomal degradation of the resulting neoantigen proteins (99). The antigen peptides generated through these processes follow two primary pathways: 1) intracellular antigens: Proteins degraded by the proteasome are further processed in the endoplasmic reticulum and loaded onto MHC class I molecules. These peptides are subsequently presented to cytotoxic T lymphocytes (CTLs), triggering a targeted immune response against tumor cells (100); 2) extracellular antigens: Proteins degraded within lysosomes are loaded onto MHC class II molecules and presented to CD4+ T cells, which play a crucial role in orchestrating the immune response (101). Additionally, activated immune cells secrete cytokines such as granzyme, perforin, TNFα, and IFNγ, which contribute to tumor cell lysis and immune-mediated tumor control.

Nucleic acid vaccines are primarily classified into DNA vaccines and RNA vaccines. These vaccines can deliver multiple tumor neoantigens in a single administration, triggering both cellular and humoral anti-tumor immune responses (102). A clinical trial utilizing linear DNA amplicons-small DNA fragments encoding neoantigens, demonstrated a significant enhancement in antigen-specific cytotoxic T lymphocyte (CTL) responses and tumor regression (103). However, DNA neoantigen vaccines face several challenges. For instance, DNA sequence introduced into the body is susceptible to degradation by nucleases, which can reduce vaccine efficacy (104).

In contrast, mRNA vaccines offer advantages such as good tolerability, lower cost, and rapid production (105). Once inside host cells, mRNA neoantigen vaccines direct ribosomes to translate the encoded genetic information they carry into neoantigen proteins (106). Compared to DNA vaccines, mRNA vaccines eliminate the risk of insertional mutations and transcriptional abnormalities, avoiding potential side effects associated with DNA integration (107). Additionally, since neoantigen proteins are synthesized through the cell’s natural translation mechanism, they may elicit a more robust immune response than peptide vaccines, which deliver pre-processed peptide segments (108). One notable example is mRNA-4157, a vaccine co-developed by Merck and Moderna, capable of encoding up to 30 different neoantigens. When administered in combination with the PD-1 inhibitor pembrolizumab, it reduced the risk of recurrence or death by 49% in patients with surgically resected high-risk melanoma (stage III/IV) and decreased the risk of distant metastasis or death by 62% (109). Personalized mRNA vaccines combined with anti-PD-1 monoclonal antibodies have also demonstrated clinical efficacy in treatmenting HNSCC. A clinical trial (NCT03468244) has reported a case in which a patient with advanced esophageal squamous cell carcinoma (ESCC)-characterized by microsatellite stability and a low likelihood of benefiting from immune checkpoint inhibitors (ICIs), achieved a partial response (PR) after treatment with mRNA personalized cancer vaccine and PD-1 monoclonal antibodies. The patient exhibited progression-free survival (PFS) of 457 days, overall survival (OS) of 457 days, and a duration of response (DOR) of 377 days (110). These findings underscore the potential of mRNA vaccines to express multiple tumor-specific neoantigens and enhance immune response when used in combination with other therapies (62). Despite these advantages, mRNA has a linear structure that is prone to degradation by RNases, resulting in relatively poor stability (111). To address this, modifications are necessary to enhance mRNA stability and further research is needed to determine optimal delivery methods and dosing strategies. Circular RNA (circRNA), which has a covalently closed-loop structure and lacks a 5’ cap and a 3’ tail, exhibits high resistance to RNases degradation and a longer intracellular half-life (112). This unique stability makes circRNA a promising candidate for next-generation vaccines. A study by Wang et al. used the permuted intron-exon (PIE) strategy to generate circRNA molecules containing internal ribosome entry site (IRES) elements and coding sequences for hepatocellular carcinoma (HCC) neoantigens. The expressed neoantigens were captured and internalized by DCs, which then presented them to CD8+ and CD4+ T cells, thereby initiating an adaptive immune response (113). DCs are considered the most potent professional antigen-presenting cells (APCs) in the human immune system (114).

Nucleic acid vaccines require the host cell’s transcription or translation machinery to synthesize antigenic proteins *in vivo*, after which antigen presentation can occur. In contrast, neoantigen peptide vaccines directly provide antigenic peptide fragments. Neoantigen peptide vaccines are the most widely used form of neoantigen-based cancer vaccines. They feature well-defined sequences, straightforward preparation and storage processes, and the ability to directly bind to MHC molecules, effectively triggering strong CD8+ T cell responses in tumors with both high and low mutation burdens (115). A clinical trial investigating a personalized neoantigen peptide vaccine for HNSCC (NCT04183166) demonstrated that in resected HPV-negative HNSCC patients, these vaccines could stimulate tumor-specific immune responses and reduce the risk of recurrence (116). The immunogenicity of neoantigen peptide vaccines can be further enhanced through the use of immunostimulatory adjuvants (117). For instance, a neoantigen peptide vaccine (NCT02897765) formulated with the polyinosinic-polycytidylic acid (poly-ICLC) as an adjuvant successfully activated CD8+ and CD4+ T cells in patients with advanced melanoma, non-small cell lung cancer (NSCLC), or bladder cancer, all of which exhibit high mutation burdens. In high-risk melanoma patients, this vaccine prevent recurrence for up to 25 months following treatment (118). Several studies have reported that neoantigen peptide vaccines are generally tolerated, with only a few cases of serious adverse events (AEs) (119). Importantly, these AEs cannot be solely attributed to the vaccines themselves but are primarily linked to cancer progression. Given their favorable safety profile and patient tolerability, personalized neoantigen peptide vaccines are regarded as a promising therapeutic approach for cancer immunology. The efficacy of neoantigen peptide vaccines is significantly influenced by peptide length, making it a critical factor in vaccine design. Short peptides typically refer to minimal peptide epitopes with optimal binding sequences. For CD8+ T cells, these epitopes consist of 8–11 amino acids, which fit within the MHC-I antigen-binding groove (120). In contrast, for CD4+ T cells, minimal peptide epitopes range from 13–18 amino acids (121). However, short peptide vaccines have a short half-life and limited immunogenicity, making it challenging to generate sustained T cell response. To overcome these limitations, many studies choose for long peptide vaccines, as they can bind to multiple HLA alleles and are more likely to induce a robust and long-lasting anti-tumor immune response (122).

As previously discussed, neoantigen vaccines based on DNA, RNA, or peptide segments typically require endogenous APCs, such as dendritic cells (DCs), to internalize the antigens and present peptide-major histocompatibility complex (pMHC) complexes to T cells (123). In this section, we focus on a distinct therapeutic approach -personalized neoantigen peptide-pulsed autologous dendritic cells vaccines. Unlike conventional vaccines, this strategy involves isolating autologous DCs from the patient, loading them *in vitro* with individualized neoantigen peptides, and subsequently reinfusing the mature, peptide-loaded DCs to stimulate a targeted T-cell response. The Neo-DCVac, a personalized neoantigen dendritic cell vaccine, is specifically designed to deliver neoantigen-loaded DCs into patients to prime the adaptive immune system. Neoantigen-pulsed DC vaccines have shown encouraging anti-tumor activity in patients with advanced or relapsed malignancies (124). In a 2024 clinical study (NCT05023928) conducted by Chen et al., the safety and feasibility of Neo-DCVac as a postoperative adjuvant treatment for esophageal squamous cell carcinoma (ESCC) were evaluated. Twelve patients were enrolled in the study, which report one- and two-year overall survival (OS) rates of 100% and 91.7%, respectively, and disease-free survival (DFS) rates of 88.3% and 66.7% (125). Despite its promising efficacy, the widespread clinical application of Neo-DCVac remains constrained by high costs, complex manufacturing protocols, and procedural risks related to leukapheresis, including vascular injury and electrolyte imbalances (126).

### 5.2 ICI combined with neoantigen-based treatment for HNSCC

Immune checkpoint inhibitors (ICIs) have demonstrated significant survival benefits in R/M HNSCC patients while maintaining a favorable safety profile. The five-year OS rate has increased from 5.0% to 15.4%-23.9% with ICI treatment (127). Before administering ICIs, PD-L1 CPS scoring, tumor mutational burden (TMB) assessment, and clinical symptom evaluation should be conducted to guide personalized treatment strategies (128, 129). The CPS (combined positive score) is reported as an integer between 0 and 100 (130). A higher CPS score correlates with an improved objective response rate (ORR) and survival benefit (31). For R/M HNSCC patients with PD-L1 CPS ≥ 1, the combined first-line treatment includes either pembrolizumab plus platinum-based chemotherapy and 5-FU or pembrolizumab monotherapy (131). For patients with unknown PD-L1 status or PD-L1 CPS < 1, the preferred first-line regimen is pembrolizumab combined with platinum-based chemotherapy and 5-FU (132). TMB has been established as a predictive biomarker for immunotherapy efficacy across multiple tumors types and serve as an indirect indicator of neoantigen generation (133). Both the 2022 ASCO guidelines and the 2023 National Comprehensive Cancer Network (NCCN) guidelines recommend pembrolizumab for first-line or later-line treatment in R/M HNSCC patients with TMB-high (≥ 10 mut/Mb) (134).

In HNSCC, combination therapy involving anti-PD-L1 and anti-CTLA-4 inhibitors demonstrates superior efficacy compared to anti-PD-L1 monotherapy, as it promotes recruitment of CD4+ T cells to tumor-draining lymph nodes (TDLN), where they differentiate into effector T cells capable of targeting and eliminating tumor cells (135). However, immune checkpoint inhibitors primarily target one or two stages of the anti-cancer immune pathway, and only a small subset of patients develop a robust anti-tumor response with single-agent therapy (67). Therefore, the combination of ICIs with personalized neoantigen vaccines has been shown to significantly enhance tumor regression compared to monotherapy (136).

Immune escape is a key mechanism driving R/M HNSCC, leading to T cell anergy and CD8+ T cell exhaustion. These exhausted T cells typically exhibit high expression of PD-1 and CD39, which suppresses the body’s ability to eliminate tumor cells through the immune system (137). Neoantigen vaccines stimulate the patient’s immune system, particularly by enhancing the response of tumor-specific CD8+ T cells (138). However, interferon-γ (IFNγ) produced by CD8+ T cells and Th1 CD4+ cells can regulate PD-L1 expression (139), which may ultimately impair the effectiveness of the vaccine by reinforcing immune suppression. To counteract this, immune checkpoint inhibitors (ICIs), including anti-CTLA-4 antibodies, anti-PD-1 antibodies, and anti-PD-L1 antibodies, bind to immune checkpoint proteins on T cells, effectively reversing tumor-mediated immune suppression and restoring T cell function (140). A study by Ott et al. demonstrated the potential of synthetic neoantigen peptide vaccines in enhancing immune responses. In this study, six melanoma patients who had undergone surgical resection were treated with neoantigen vaccines, followed by PD-1 antibody therapy. Among them, two patients achieved complete tumor regression, highlighting the synergistic effect of neoantigen vaccines and ICIs (118). Several ongoing clinical trials are investigating personalized neoantigen vaccines in combination with immune checkpoint inhibitors for HNSCC treatment. Examples include PANDA-VAC (NCT04266730), PGV002 (NCT05192460), and NeoDC-Vac (NCT06675201), as detailed in **Table 1**.

## 6 Conclusion

This review provides an overview of HNSCC treatment strategies targeting tumor neoantigens and presets theoretical evidence supporting the clinical relevance of neoantigen-based immunotherapies. However, personalized neoantigen vaccines for HNSCC remain limited in both market availability and clinical development. A primary challenge lies in the screening and identification of highly immunogenic neoantigens, which heavily rely on high-throughput sequencing and bioinformatics techniques. Technical inaccuracies in these processes may result in false positives, leading to the selection of ineffective neoantigens that fail to elicit robust immune responses (141). Neoantigen identification can be enhanced by employing computational algorithms to construct virtual peptidomes from NGS data, combined with mass spectrometry(MS)-based analysis of peptides bound to MHC molecules. Integrating genomic and transcriptomic sequencing data with HLA-associated peptideome analysis has further improved the sensitivity and specificity of neoantigen identification (142). For clinical applications, an efficient computational workflow is essential for precise neoantigen selection. Despite rapid advances in sequencing technologies, neoantigen identification and validation remain time-consuming and costly. The process of developing neoantigen vaccines from patient tumor samples typically requires three to five months (108), significantly limiting their clinical feasibility.

By targeting PD-L1 overexpression, CD58 genetic alterations, and the immunosuppressive microenvironments, more effective combination treatment strategies can be designed, potentially improving the prognosis of patients with HNSCC. In diffuse large B-cell lymphoma (DLBCL), translocation of the PD-L1 gene locus with the IGH gene frequently results in PD-L1 overexpression (143). Similar overexpression of PD-L1 has been observed in HNSCC. Future studies investigating the genetic basis for PD-L1 upregulation in HNSCC could benefit from methodologies previously employed in DLBCL research. Elevated PD-L1 expression is associated with tumor aggressiveness and poor clinical outcomes; accordingly, combined blockade of PD-L1 and CD73 has demonstrated significant inhibition of tumor growth and metastasis (144). While PD-L1 inhibitors have shown clinical efficacy in treating HNSCC, their combination with CD73 inhibitors or other immune checkpoint inhibitors may further enhance antitumor immune responses. Furthermore, CD58 has been shown to suppress PDL-1 and IDO expression by inhibiting the JAK2/STAT1 pathway through activation of the LYN/CD22/SH2 domain-containing phosphatase 1 (SHP1) axis (145). Therefore, combining PD-L1 inhibitors with approaches designed to enhance CD58 signaling may offer a promising strategy to overcome PD-L1-mediated immune suppression (146).

However, neoantigen vaccines alone are unlikely to achieve complete tumor eradication (147). First, tumors with low TMB might be unsuitable for existing neoantigen vaccine strategies. For example, nasopharyngeal carcinoma exhibits a lower mutation rate of only 1 mutation/Mb (148). This relatively low rate complicates the identification of immunogenic neoantigens. Second, immune escape mechanisms in tumors remain a significant obstacle to the efficacy of tumor vaccines (149). Combining neoantigen vaccines and ICIs offers a promising strategy to overcome tumor immune evasion (150). However, research on neoantigen vaccine-ICI combination therapy is still in the clinical trial phase (102), requiring further investigation and clinical validation to establish its safety and efficacy. With continued scientific advancements and technological progress, the widespread clinical adoption of neoantigen-based immunotherapy-particularly in HNSCC is expected. Future developments may lead to enhanced treatment efficacy, reduced preparation time, and greater accessibility, ultimately improving patient outcomes.
